# Unveiling Rarity: A Case Report of a Poorly Differentiated Synovial Sarcoma Mimicking a Plunging Ranula

**DOI:** 10.7759/cureus.64290

**Published:** 2024-07-10

**Authors:** Jorge Sanchez, Nehemias Guevara, Volha Chapiolkina, Esmirna M Perez Rosario, Maria C Tole, Yemesrach Mekonen, Ilmana Fulger

**Affiliations:** 1 Internal Medicine, St. Barnabas Hospital Health System, New York, USA; 2 Hematology-Oncology, St. Barnabas Hospital Health System, New York, USA

**Keywords:** surgical margins, immunohistochemistry, sarcoma soft tissue, head and neck tumors, synovial sarcoma

## Abstract

Synovial sarcomas are uncommon and highly aggressive sarcomas. Typically, they start in the soft tissues of the extremities, although they may develop in the head and neck region in rare cases. When they do, they usually present with localized symptoms in the affected area. Our patient is a 20-year-old man without a medical history who complained of a three-month history of submental swelling of the left side with a non-tender, palpable 5 cm mass. Initially believed to be a plunging ranula, the patient underwent transoral excision of the left submandibular soft tissue mass in the neck by the ear, nose, and throat (ENT) specialist. The pathological analysis of the mass confirmed the presence of a poorly differentiated synovial sarcoma. A postoperative neck imaging was performed, which showed a significant decrease in mass size compared to the previous imaging; however, the mass was still present. This is one of the few described cases of a poorly differentiated synovial sarcoma located on the floor of the mouth. Therefore, it highlights the importance of considering it as a possible differential diagnosis of head and neck pathologies.

## Introduction

Sarcomas are a diverse group of malignancies originating from connective tissue, such as fat, muscle, blood vessels, bone, and cartilage. Conventionally, they are classified based on their morphology and the tissue they histologically resemble. For example, a tumor that usually resembles skeletal muscle is called rhabdomyosarcoma [1].

The cell of origin of various sarcomas is unclear but believed to result from genetic mutations in their mesenchymal progenitors. Fortunately, in these cases, molecular genetic analysis has significantly helped identify sarcomas and can further distinguish them by genetic subtypes [1].

Synovial sarcomas are a rare and aggressive type of sarcoma that accounts for approximately 10% of all soft tissue sarcomas. Usually originating from soft tissue in the extremities, they can arise from the head and neck on rare occasions, with localized symptoms (due to compression) based on the organ of involvement [2]. However, they are usually asymptomatic in their early stages, allowing them to progress to more advanced stages before the patient receives medical attention.

Unfortunately, just as in this case report, even if identified early enough and surgery is performed, the resection may not be complete, requiring more aggressive treatments despite early intervention in the disease course.

## Case presentation

A 20-year-old male without a medical history but a social history of occasional hookah use presented to the ENT (ear, nose, and throat) clinic for a three-month history of left-sided submental swelling associated with an elevation of the floor of the mouth and a palpable non-tender mass. At the time, the patient denied fever, night sweats, and weight loss. Initially, this was concerning for a salivary ductal stone, an infected cyst, or a plunging ranula.

**Figure 1:**
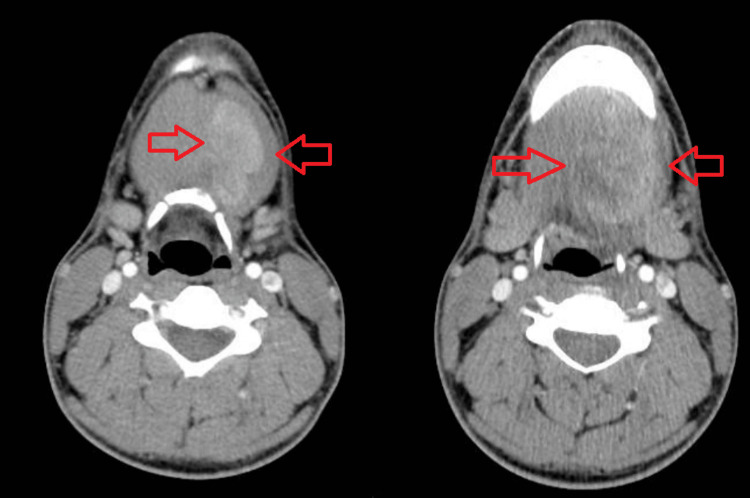
CT scan of the neck with intravenous contrast enhancement. Two different radiographic cuts: the higher cut and the lower cut, progressing from right to left.

A computed tomography (CT) scan of the neck's soft tissue revealed a heterogeneous enhancement, a mass-like lesion measuring 6.8 x 3.8 x 5.4 cm involving the left sublingual space, with intrinsic septations and hypodensity areas. It effaced the left side of the piriform sinus and narrowed the airway with a shift from left to right. There was no cervical or retropharyngeal lymphadenopathy (Figure 1).

Initially believed to be a plunging ranula, the patient underwent transoral excision of the left submandibular soft tissue mass in the neck by ENT. The gross specimen consisted of multiple fragments of light gray to pink soft tissue mass that measured 8 x 5 x 3 cm in aggregate.

**Figure 2:**
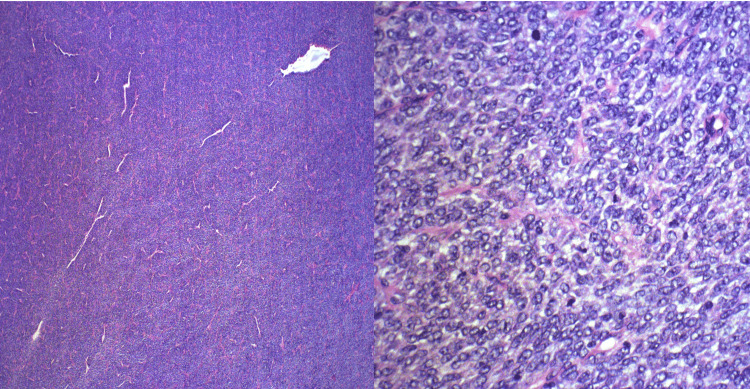
The low-power objective is positioned on the right slide, providing a total magnification of 40×, while the high-power objective is located on the left slide, offering a total magnification of 400× of the tumor.

Microscopy showed a large, encapsulated spindle cell neoplasm with a prominent hemangiopericytic architecture involving all peripheral surfaces. The tumor was cellular and composed of a mixture of fascicular and nested areas. In the fascicular regions, the neoplasm was composed of uniform spindle cells with scant cytoplasm with indistinct borders and oval nuclei with granular chromatin. Tumor cells were more rounded in nested areas, with vesicular chromatin and occasionally conspicuous nucleoli. In these latter areas, tumor cells showed a higher mitotic rate with up to 30 mitoses per 1 mm^2 ^(Figure 2).

Immunohistochemistry (IHC) was strongly positive for the synovial sarcoma X chromosome gene (SSX) and serine S818 (S818). Pankeratin and epithelial membrane antigen (EMA) showed patchy and weak positivity in lower-grade fascicular areas. S100 (a family of cytoplasmic calcium-binding proteins) stains scattered individual cells in these areas. CD34 (a transmembrane glycoprotein) highlighted the rich vasculature, and the signal transducer and activator of transcription 6 (STAT6) were negative. Ki-67 (a protein that is found only in cells that are dividing) showed variable staining, ranging from 20%-30% of tumor cells in the fascicular areas up to 70% in the poorly differentiated areas.

The findings aligned with a poorly differentiated synovial sarcoma, extensively involving all surfaces with positive margins. The diagnosis of synovial sarcoma was confirmed by using an SYT (SS18) (18q11.2) break-apart fluorescence in situ hybridization (FISH) probe that detects a synovial sarcoma translocation in chromosome 18.

Postoperative neck imaging was performed, which showed a significant decrease in the mass size compared to the previous one, but it was still present. A positron emission tomography scan (PET/CT) showed a mass on the left part of the floor of the mouth, but there was no evidence of nodal disease or distant metastatic disease.

The patient will undergo neoadjuvant chemotherapy with ifosfamide/doxorubicin alternating with ifosfamide for a total of four cycles as described by the ARM D of ARST0332 study from the Children’s Oncology Group, followed by primary site surgery with an attempt to achieve complete resection with negative margins. The patient will then receive adjuvant chemotherapy with AIM protocol (consisting of Adriamycin, ifosfamide, and mesna) alongside radiation therapy.

## Discussion

Synovial sarcomas are a subtype of soft tissue sarcomas. The word "synovial" is misleading because the tumor can originate anywhere in the body and is not limited to the synovium [2]. It is believed to originate initially from undifferentiated mesenchymal cells [3]. This type of sarcoma is further classified by its histology as monophasic, biphasic, and the rare variation of poorly differentiated [2,4].

The monophasic type of synovial sarcoma is characterized by uniform spindle or epithelial cells. The biphasic type has epithelial cells arranged in glandular structures or spindle cells arranged in fascicles, while the poorly differentiated type has round cells [2,4].

Synovial sarcomas account for approximately 5% to 10% of all soft tissue sarcomas, most commonly seen in young adults, and are generally found in the lower extremities (46.1% of the time) [5]. They present as a deep-rooted growing mass, hard to palpate, not tender, and minimally movable within the muscle [2]; they are rarely located in the head and neck, not to mention the oral cavity, and tend to be aggressive in such cases with a high potential to metastasize early [2,6].

Synovial sarcoma is not typically associated with environmental or hereditary factors. While radiation-induced synovial sarcoma is exceedingly rare, it has been documented in the medical literature [2].

In the United States, approximately 800-1000 new cases of synovial sarcoma are diagnosed each year. According to an analysis of the Surveillance, Epidemiology, and End Results (SEER) database, the age-adjusted incidence rate of synovial sarcoma in the US is approximately 0.177 per 100,000 people, equating to approximately 580 incident cases annually. The prevalence rate is estimated at 0.65 per 100,000, indicating there are approximately 2129 prevalent cases of synovial sarcoma in the US [2].

The vast majority of this type of sarcoma (> 95%) has an SS18-SSX fusion gene, which serves as a clinical diagnostic marker [7], as in the presented case.

Treatment strategies for synovial sarcoma are dictated by the disease stage at diagnosis, which can vary from localized to metastatic. In cases of localized disease, surgical resection is typically pursued to achieve microscopic negative margins (R0 resection) [2].

Excision of the complete tumor with negative margins is considered the optimal primary treatment for early-stage local disease [8]. For instance, Rao et al. [9] reported successful tumor resection with clear surgical margins, and no recurrence was observed after one year.

Patients with locally advanced unresectable or metastatic synovial sarcoma are managed with therapies aimed at palliative care and prolonging survival. These approaches may include cytotoxic chemotherapy, tyrosine kinase inhibitors, or participation in clinical trials [2].

Unfortunately, as in our case report, total excision is sometimes impossible due to the anatomical location and advanced disease at the moment of the presentation. In such cases, an approach with multiple therapies is usually the treatment, such as a combination of chemotherapy, followed by surgery, and then postoperative radiation [8,10].

Diagnosing synovial sarcoma clinically poses significant challenges. However, a high index of suspicion for synovial sarcoma is crucial when evaluating a previously healthy young patient, typically in the adolescent or young adult age group, who presents with a firm mass in the extremities or elsewhere in the body [2].

One significant reason why surgical margins are often positive after resection of synovial sarcomas, as observed in our case, is their frequent radiographic misclassification as other benign structures such as cysts or fibroadenomas. This misidentification, as illustrated in the case by Wang et al. [3], often leads to initial assumptions that may delay biopsy until after resection.

Krieg et al. describe the five-year survival rate in patients with synovial sarcoma as 75.8% and the 10-year survival rate as 62.9% [10,11]. However, the 15-year survival rate was only 46.5% [10,11], which could reflect how late a metastasis finding is discovered in these patients. The authors suggested follow-up periods of >10 years with good patient education, detailed history, and physical examination to detect early recurrence [6,10,11].

This case is doubly rare due to its histological type (poorly differentiated synovial sarcoma) and its location in the oral cavity, specifically on the floor of the mouth, making this type of sarcoma a rarer and more aggressive form.

## Conclusions

The case underscores the complexity and challenges of diagnosing and treating poorly differentiated synovial sarcomas, particularly when they occur in rare locations such as the oral cavity. These tumors often present ambiguously, resembling benign structures, as in our case, which complicates disease management. The literature extensively discusses the limitations of radiology in accurately categorizing soft tissue tumors. This emphasizes the need for a low threshold when performing diagnostic fine needle aspiration cytology (FNAC) to establish preoperative tissue diagnoses, especially when the primary diagnosis is uncertain.

Furthermore, while advances in treatment modalities have improved the prognosis for synovial sarcomas, the aggressive nature of poorly differentiated variants necessitates a multidisciplinary approach integrating surgery, chemotherapy, and radiation. Therefore, clinicians must remain vigilant regarding this aggressive cancer type to facilitate prompt diagnosis and implement a comprehensive treatment strategy.
